# Pathogens as Predictors of Honey Bee Colony Strength in England and Wales

**DOI:** 10.1371/journal.pone.0133228

**Published:** 2015-07-17

**Authors:** Giles E. Budge, Stéphane Pietravalle, Mike Brown, Lynn Laurenson, Ben Jones, Victoria Tomkies, Keith S. Delaplane

**Affiliations:** 1 The Food and Environment Research Agency, Sand Hutton, York, YO411LZ, United Kingdom; 2 Department of Entomology, University of Georgia, Athens, GA, 30602, United States of America; University of North Carolina, Greensboro, UNITED STATES

## Abstract

Inspectors with the UK National Bee Unit were asked for 2007-2008 to target problem apiaries in England and Wales for pathogen screening and colony strength measures. Healthy colonies were included in the sampling to provide a continuum of health conditions. A total of 406 adult bee samples was screened and yielded 7 viral, 1 bacterial, and 2 microsporidial pathogens and 1 ectoparasite (*Acarapis woodi*). In addition, 108 samples of brood were screened and yielded 4 honey bee viruses. Virus prevalence varied from common (deformed wing virus, black queen cell virus) to complete absence (Israeli acute paralysis virus). When colonies were forced into one of two classes, strong or weak, the weak colonies contained more pathogens in adult bees. Among observed pathogens, only deformed wing virus was able to predict colony strength. The effect was negative such that colonies testing positive for deformed wing virus were likely to have fewer combs of bees or brood. This study constitutes the first record for *Nosema ceranae* in Great Britain. These results contribute to the growing body of evidence linking pathogens to poor honey bee health.

## Introduction

Since the middle of the last decade honey bee decline has galvanized a multi-national effort to understand the extent of the problem, characterize its causative or associated factors, and develop mitigating practices and policies. The problem is concentrated in the Northern Hemisphere where winter losses are reported from around 30% in the USA [1], 10–85% in the Middle East [2], and 1.8–53% across Europe [2,3]. Colony losses are not confined to winter and may reach 20% in summer in the USA [4]. A metagenomic analysis from the winter of 2006–2007 showed that virtually all bees from collapsing colonies carried numerous pathogens [5], and subsequent work has substantiated that bee decline is a complex of interacting drivers both biotic and abiotic [6,7,8,9], although depredation by the parasitic mite *Varroa destructor* is a common thread wherever high bee morbidity is reported [10,11,12,13,14].

This paper is part of a growing body of evidence that associates specific risk factors with colony death or decline. These papers vary in the case definitions used to categorize colonies that are healthy or unhealthy, the risk categories considered, and the geographic ranges included [6,7,12,14,15,16,17,18]. In the present study we focus on pathogens as predictors of colony decline and death in England and Wales. Sampling across these countries was completed by inspectors of the UK National Bee Unit (NBU) who are active between April-October [19]. For the present paper we collated colony strength data with pathogen profiles in colonies across England and Wales for the years 2007 and 2008. We eliminated colonies from the study for which the cause of death was traceable to obvious factors such as starvation, queen failure, uncontrolled *V*. *destructor* mites or acute pesticide kill, leaving pathogens as the risk factors of interest. Our broad goal was to characterize the pathogen profiles that best predict colony decline in this region.

## Materials and Methods

### Long-term monitoring of honey bee colony losses

**Fig 1:**
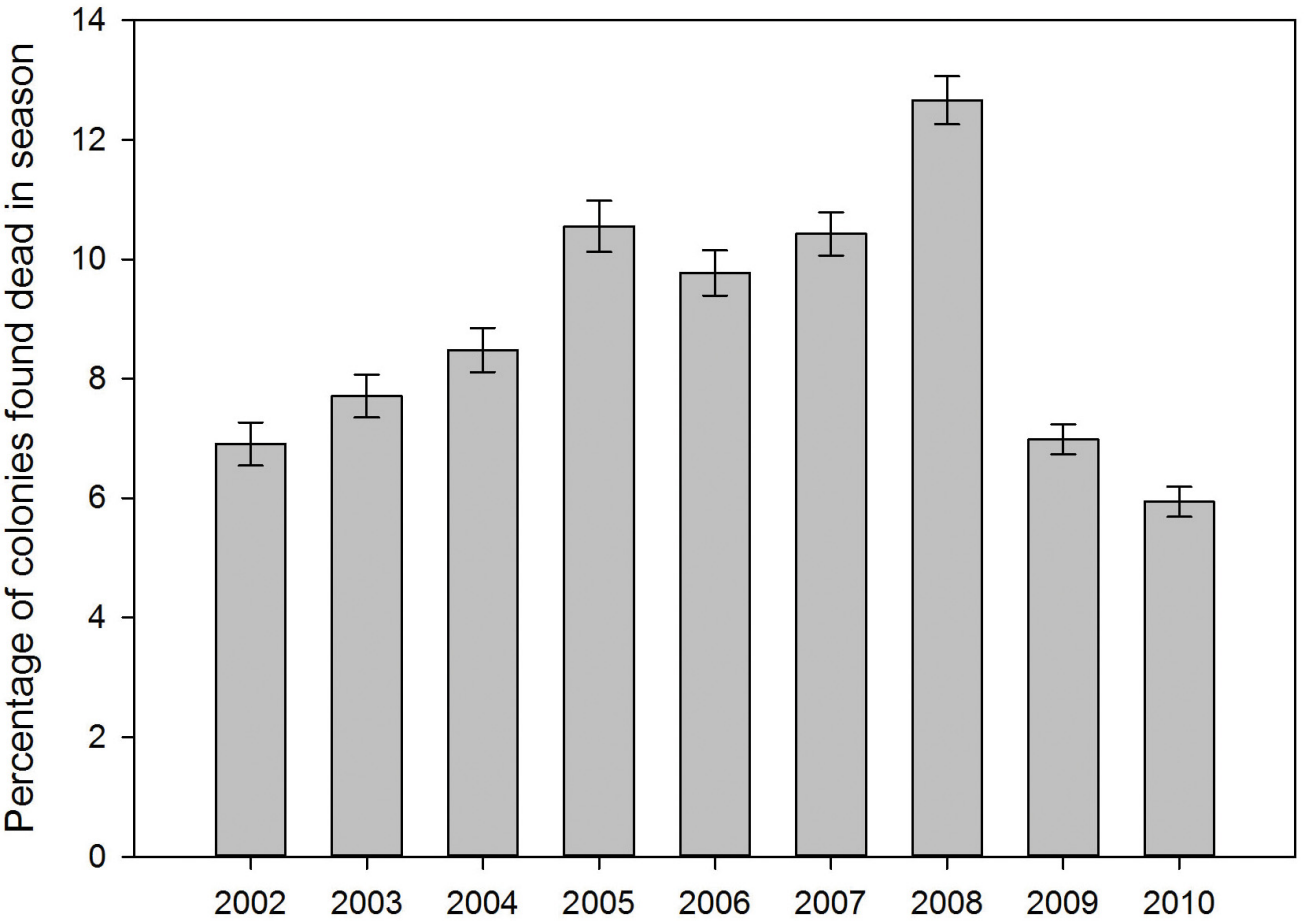
The annual percentage of colonies found dead during the inspection season (Apr-Oct) for 2002–2010.

The monitoring of honey bee health is performed by a government funded apiary inspection programme in England and Wales, operated by the National Bee Unit (NBU; http://www.nationalbeeunit.com). NBU inspectors routinely record the number of colonies found dead during each inspection. The annual percentage of colonies found dead during the inspection season (Apr-Oct) for 2002–2010 are shown in Fig 1 to give context to the current study.

### Sampling for pathogen screens

For each of the two years 2007 and 2008, NBU inspectors singled out colonies with significant problems for retrospective sampling and analysis. Cases of colony death traceable to obvious explanations, e.g., starvation, queen failure, or poor *V*. *destructor* control, were first eliminated with a triage process. From among the remaining colonies in the apiary, alive or dead without traceable explanation, samples of bees and brood were collected in 70% ethanol and stored for pathogen analysis. Larvae were sampled at ages 1–6 days post-eclosion. Measures of colony strength were taken by counting the number of combs of adults bees and brood present in each colony [20], assigning values of zero for colonies that were dead. For 2007, 72 adult bee samples were analysed for infestation by the tracheal mite, *Acarapis woodi*; each bee was dissected and the main thoracic tracheal trunks inspected microscopically for evidence of mites or their damage.

### Pathogen screening

The nucleic acid was recovered from bulk samples of adult bees and brood samples (*n* = 5 bees per sample [2007]; *n* = 30 bees [2008]) and tested using PCR or real-time RT PCR as appropriate. This sample size disparity is explained by improved analytical capacity in 2008; it is also the reason why subsequent statistical analyses were run by year. DNA extracts were prepared from adult bees by grinding to homogeneity in a ‘long special universal’ bag using a Lenze grinder (Bioreba). DNA was extracted from cleared lysate using a Wizard Magnetic DNA Purification System for Food (Promega, FF3750) in conjunction with a Kingfisher ML magnetic particle processor (Thermo Electron Corporation) as described by Ward et al. [21]. RNA extracts from brood and adult bees were prepared using a Guanidine isothiocyanate lysis buffer in combination with a Kingfisher ML magnetic particle processor [22]. All nucleic acid extracts were stored at -80°C prior to analysis. Brood samples were screened for eight honey bee viruses: Acute bee paralysis virus (ABPV), *Apis* iridescent virus (AIV), black queen cell virus (BQCV), chronic bee paralysis virus (CPBV), deformed wing virus (DWV), Israeli acute paralysis virus (IAPV), Kashmir bee virus (KBV), and sacbrood virus (SBV). Adult bee samples were screened for *Nosema apis* (*N*. *apis F1* –ATT TAC ACA CCA GGT TGA TTC TGC; *N*. *apis R1* –TGA GCA GTC CAT CTT TCA GTA CAT AGT; *N*. *apis probe*–MGB-TGA CGT AGA CGC TAT TC), *N*. *ceranae* (*Nosema c1 836F*—TTG AGA GAA CGG TTT TTT GTT TGA G; *Nosema c1 974R*—TTC CTA CAC TGA TTG TGT CTG TCT TTA A; *Nosema c1 865T* –FAM-ATA ATA GTG GTG CAT GGC CGT TTT CAA TGG-TAMRA) and *Melissococcus plutonius* [23] in addition to all 8 honey bee viruses [22]. Assay *AJ307465-955F/1016R/975T*, designed to *Apis mellifera* 18S rRNA [21], was used as an internal control to demonstrate that extractions were of sufficient quality for product amplification.

All TaqMan assays were set up using PCR Core Reagent Kits (PE Biosystems Cat. No. 4304441) consisting of 1 × buffer A, 0·025-U μL–1 AmpliTaq Gold, 0·2 mM dNTPs and 5·5 mM MgCl2. All sets of primers were used at 300 nM and probes at 100 nM with 10 μL of template, giving a final volume of 25 μL per reaction. Reactions were carried out within the ABI Prism 7900HT Sequence Detector System (PE Biosystems) beginning with 50°C for 2 min, 95°C for 10 min, and 40 cycles of 95°C for 15 s and 60°C for 1 min. RT PCR reactions were identical to those for PCR except for the addition of 0.2U MMLV (Fermentas) and the addition of a 30 min RT step at 48°C (Kajobe et al., 2010). The *C*_T_ value for each reaction was assessed using Sequence Detection Software v2.2.2 (Applied Biosystems). Samples were deemed positive when both duplicate reactions had a *C*_T_ value less than 40 [23].

### Statistical analyses

Response variables (number of combs of bees or brood) were analyzed by year (2007 or 2008) and by material sampled (adults bees or brood). First, we classified each colony as “strong” if it had >5 combs of bees or “weak” if it had ≤5 combs of bees (dead colonies were assigned values of zero). In exploratory epidemiological work, including honey bee disorders, it is common to assign subjects into such “case definitions” as strong vs. weak, well vs. unwell, then work retrospectively to find predictors of the condition [6,7,17,24,25]. We then used a one-way analysis of variance to determine how the mean numbers of pathogens detected in colony samples differed between the two strength classes. Secondly, to screen for predictors of colony strength we used multiple linear regression to test the extent to which the number of combs of brood varied as a function of the presence or absence of brood pathogens and the number of combs of adults varied as a function of presence or absence of adult pathogens. All analyses were done with Genstat version 15.1.

## Results

### Long-term monitoring of honey bee colony losses

During 2002–2010, between 19,044–39,463 colonies were inspected annually. The highest number of colony losses reported in any one year was 3,253. The two years of this study, 2007–2008, constituted a period of high colony mortality immediately preceding an improving trend in 2009–2010 (Fig 1).

### Pathogen screening

The triage procedure yielded data on known or suspected causes of colony death for the two years of intense sampling, 2007 and 2008, and are shown in Table 1. NBU inspectors most frequently implicated *V*. *destructor* and “failed queens.” Relatively few colonies were found with symptoms of acute pesticide poisoning, and subsequent chemical analysis suggested significant chemical residues in only 2 colonies from 2007 (actives implicated: bendiocarb and dieldrin) and 5 colonies from 2008 (bendiocarb, bifenthrin, permethrin) [26]. The most conspicuous result here is the high numbers of colony deaths for which these experienced bee handlers could offer no field diagnosis.

**Table 1:** Known or suspected causes of colony death in two years of concentrated sampling for this study. The total number of colonies inspected each year is also provided.

Year	Suspected reason for colony death				Total number colonies dead	Total colonies inspected
	Varroosis	Failed queens	Starvation	Acute pesticide kill		
2007	137	16	22	19	2,776	26,648
2008	434	141	85	27	3,342	26,391

The number of positive adult bee and brood samples for each tested pathogen is given in Table 2 along with values for (1) positive predictability (percentage of samples testing positive for pathogen in weak colonies), (2) sensitivity (percentage of weak samples testing positive for pathogen), and (3) specificity (percentage of strong samples testing negative for pathogen). A total of 406 adult bee samples (254 in 2007, 152 in 2008) was screened and yielded 7 viral, 1 bacterial, and 2 microsporidia pathogens and 1 ectoparasite (*Acarapis woodi*). In addition, 181 samples of brood (101 in 2007, 80 in 2008) were screened and yielded 4 honey bee viruses. Virus prevalence varied from common (DWV, BQCV) to complete absence (IAPV). Adult bees tended to contain more viruses than brood; indeed ABPV and KBV were only found in adults.

**Table 2:** Targeted screening for known honey bee pathogens using PCR or RT PCR as appropriate. Tabular values outside of parentheses are for adult bees, and values inside parentheses are for brood. For 2007, total number of samples for bees was 254 (115 weak, 139 strong), and total number for brood was 101 (33 weak, 68 strong). For 2008, total number of samples for bees was 152 (89 weak, 63 strong), and total number for brood was 80 (28 weak, 52 strong). Calculations for column parameters are as follows:
$$Proportion\;of\;weak\;samples\;positive=\frac{Number\;positive\;and\;weak}{Number\;weak}$$
$$Proportion\;of\;strong\;samples\;positive=\frac{Number\;positive\;and\;strong}{Number\;strong}$$
$$Positive\;predictor=\frac{Number\;positive\;and\;weak}{Number\;positive}$$
$$Sensitivity=\frac{Number\;positive\;and\;weak}{Number\;weak}$$
$$Specificity=\frac{Number\;non-positive\;and\;strong}{Number\;strong}.$$

	Weak colonies (≤5 combs of bees)		Strong colonies (>5 combs bees)				
Pathogen	No. positive samples	% positive	No. positive samples	% positive	Positive predictor	Sensitivity	Specificity
2007							
DWV	65 (17)	56.5 (51.5)	47 (8)	33.8 (11.8)	58 (68.0)	56.5 (51.5)	66.2 (88.2)
*N*. *ceranae*	54	47	61	43.9	47	47	56.1
*N*. *apis*	23	20	15	10.8	60.5	20	89.2
BQCV	21 (1)	18.3 (3.0)	25 (3)	18 (4.4)	45.7 (25.0)	18.3 (3.0)	82 (95.6)
CBPV	19	16.5	22	15.8	46.3	16.5	84.2
*A*. *woodi*	11	9.6	13	9.4	45.8	9.6	90.6
SBV	5	4.3	5	3.6	50	4.3	96.4
KBV	1	0.9	0	0	100	0.9	100
ABPV	0	0	1	0.7	0	0	99.3
AIV	0	0	0	0	-	0	100
IAPV	0	0	0	0	-	0	100
2008							
DWV	71 (19)	79.8 (67.9)	31 (21)	49.2 (40.4)	69.6 (47.5)	79.8 (67.9)	50.8 (59.6)
BQCV	57 (16)	64 (57.1)	37 (19)	58.7 (36.5)	60.6 (45.7)	64 (57.1)	41.3 (63.5)
*N*. *apis*	48	53.9	37	58.7	56.5	53.9	41.3
*N*. *ceranae*	32	36	21	33.3	60.4	36	66.7
SBV	5 (1)	5.6 (3.6)	1 (1)	1.6 (1.9)	83.3 (50.0)	5.6 (3.6)	98.4 (98.1)
*M*. *plutonius*	6	6.7	1	1.6	85.7	6.7	98.4
CBPV	2 (1)	2.2 (3.6)	1 (1)	1.6 (1.9)	66.7 (50.0)	2.2 (3.6)	98.4 (98.1)
KBV	1	1.1	1	1.6	50	1.1	98.4
APBV	0	0	1	1.6	0	0	98.4
AIV	0	0	0	0	-	0	100
IAPV	0	0	0	0	-	0	100

When colonies were forced into one of two classes, strong (>5 combs of bees) or weak (≤5 combs of bees), the weak colonies contained significantly more pathogens in adults (Table 3). Among the suite of pathogens sampled, DWV emerged as the only pathogen in multiple linear regression with power to predict colony strength. The effect was consistently negative such that DWV positive colonies were likely to have fewer combs of bees or brood. This regression result is consistent with the uniformly high values DWV shows for positive predictability, sensitivity, and specificity compared to other viruses (Table 2). When colonies were forced into classes of DWV present or absent, the presence of DWV was correlated to a significant reduction in the number of combs of bees (Table 4) and brood (Table 5), although the effect was only suggestive (*P* = 0.061) for brood samples in 2008 (Table 5). When samples from dead colonies were removed from analyses we found that DWV remained a predictor for poor colony strength. Samples of adult bees from dead colonies numbered 21 out of 254 in 2007 and 39 out of 152 in 2008. Samples of brood from dead colonies numbered 4 out of 101 in 2007 and 2 out of 80 in 2008.

**Table 3:** Mean number (± 95% CI) colony pathogens detected in samples of adult bees. 2007 test of means: *F* = 5.76; df = 1,252; *P* = 0.017. 2008 test of means: *F* = 4.61; df = 1,150; *P* = 0.033.

	Weak colonies			Strong colonies		
	Lower CI	Mean (*n*)	Upper CI	Lower CI	Mean (*n*)	Upper CI
2007	1.51	1.73 (115)	1.96	1.15	1.36 (139)	1.56
2008	2.25	2.49 (89)	2.74	1.79	2.08 (63)	2.37

**Table 4:** Mean number (± 95% CI) of combs of bees as predicted by presence or absence of deformed wing virus. 2007 test of means: *F* = 16.93; df = 1,252; *P*<0.001. 2008 test of means: *F* = 21.39; df = 1,150; *P*<0.001.

	DWV absent			DWV present		
	Lower CI	Mean (*n*)	Upper CI	Lower CI	Mean (*n*)	Upper CI
2007	6.54	7.56 (142)	8.65	3.73	4.61 (112)	5.58
2008	5.53	7.74 (50)	10.32	1.56	2.4 (102)	3.43

**Table 5:** Mean number (± 95% CI) of combs of brood as predicted by presence or absence of deformed wing virus. 2007 test of means: *F* = 20.89; df = 1,99; *P*<0.001. 2008 test of means: *F* = 3.62; df = 1,78; *P* = 0.061.

	DWV absent			DWV present		
	Lower CI	Mean (*n*)	Upper CI	Lower CI	Mean (*n*)	Upper CI
2007	2.99	3.55 (76)	4.16	0.80	1.36 (25)	2.06
2008	3.18	4.18 (40)	5.32	2.04	2.86 (40)	3.81

## Discussion

Compared to strong colonies, weak colonies were more frequently associated with higher numbers of pathogens in adult bees (Table 3), a trend shown before by investigators in the USA [6,7] and Belgium [14]. This interesting finding suggests that the presence of our principle predictor (DWV) might reduce the ability of honey bees to respond to challenge from other pathogens. Whilst only one of several possible explanations, this would be consistent with the observation that DWV induces an immune-suppressive syndrome in bees from weak colonies [27], thereby allowing other pathogens to replicate to detectable levels. Among the pathogens detected in the present study, only DWV was shown to predict colony strength such that DWV positive colonies were likely to have fewer combs of bees or brood. When the DWV data were examined categorically—DWV present or absent—colonies positive for DWV had fewer combs of bees (Table 4) and brood (Table 5) than negative colonies. The size of honey bee colonies and the prevalence of honey bee pathogens like DWV [27] fluctuate seasonally. In our study, samples were collected as the need to investigate colony losses arose. As such, seasonality was not a fixed element of design, and so it is possible that our observed links between DWV and small colony size is an artefact of coincidental timings of seasonal fluctuations between host and pathogen. This hypothesis seems unlikely however, given that colonies with adult bees testing positive for DWV were smaller than those testing negative for DWV in 12 of 14 months, and these included May-August, when honey bee colonies are typically at their largest in the UK (data not shown).

It is important to note that these significant differences were detectable in a data cloud with a large amount of random variation. These large and spurious effects include (1) a wide geography from over 400 apiaries across England and Wales, (2) from 52 participating beekeepers, (3) 30 NBU inspectors making subjective strength determinations across a range of (4) non-standardized comb dimensions: beekeepers in the UK use no fewer than 7 hive dimensions [28]. In spite of these sources of random noise, the significant effects of pathogens were detectable.

Our results are consistent with overriding conclusions drawn by other teams searching for pathogens that predict honey bee decline. Foremost, our study substantiates the importance of DWV. This pathogen has been among the most implicated predictors of bee decline from surveys in Germany [16], Greece [15], the United States [7], Switzerland [12], and (for various pollinators including honey bees) the UK [18]. In contrast, DWV was not among the most important predictors in similar studies from the USA [6] and Belgium [14]. Given the strong association of DWV with the pandemic ectoparasite *V*. *destructor* [8,29] the predominance of DWV should not be surprising. However it is worth noting that whilst our study did not measure mite infestation levels in the colonies sampled, the triage procedure had eliminated colonies for which varroosis was clinically detectable at the time of sampling. Even though DWV levels are expected to fluctuate with *Varroa* levels [30], it is possible our results indicate a legacy effect of DWV persisting in spite of effective mite treatments, as suggested by vanEngelsdorp et al. [6] for colonies expressing symptoms of colony collapse disorder (CCD). Alternatively, these data may suggest a shift in the ability of DWV to affect colony health. RNA viruses are known to evolve rapidly due to high mutation rates, high yields, and short replication times [31]. Perhaps DWV has evolved the ability to cause damage in the absence of *Varroa*. More experiments are required to test both hypotheses.

IAPV and KBV have been identified as significant risk factors in the development of CCD in the USA [5,7]. Interestingly, NBU inspectors reported no colonies suffering from CCD-like symptoms, and these sister Dicistroviruses were rarely found in this survey, suggesting low prevalence in England and Wales.

The microsporidian *N*. *ceranae* has been implicated in colony losses in Spain [17,32], Greece [15], and Belgium [14]. The present study constitutes the first record of *N*. *ceranae* in Great Britain; however its congener *N*. *apis* was more frequently detected than *N*. *ceranae* in adult bees. Neither microsporidian was significantly associated with poor colony strength, supporting a premise that the virulence of these pathogens is variable and region-specific [33]. Viral pathogens were more frequently detected in adult bee samples than in brood. Considered along with the ability to test for DNA-diagnosed disorders like *Nosema* spp. or the ectoparasite *Acarapis woodi*, these data suggest adult bees may be particularly useful in future surveys interested in determining pathogen prevalence.

Our results suggest that beekeepers in England and Wales should concentrate on *Varroa* control, queen maintenance, and timely feeding to reduce colony losses. A clear link was demonstrated between poor colony strength and presence of DWV. Owing to the tight link between *Varroa* and DWV, and until effective anti-viral products are available to combat this virus, the only recourse available to beekeepers is to control *Varroa* as a proxy for controlling DWV. Pathogens are a significant fraction of the causes contributing to bee decline, not just from known diseases like Nosemosis, but also from emerging pests and pathogens outside of our normal consideration, as was shown with the trypanosomatid bee parasite *Crithidia mellificae* in Belgium [14]. Long term data sets are key to advancing our understanding of the comparative impacts of biotic and abiotic factors in honey bee colony health.
